# The IL-17-IL-17RA axis is required to promote osteosarcoma progression in mice

**DOI:** 10.1038/s41598-023-49016-1

**Published:** 2023-12-07

**Authors:** Naoto Yoshimura, Ryusho Kariya, Masaki Shimada, Makoto Tateyama, Hideto Matsunaga, Yuto Shibata, Shuntaro Tanimura, Kosei Takata, Takahiro Arima, Junki Kawakami, Kazuya Maeda, Yuko Fukuma, Masaru Uragami, Katsumasa Ideo, Kazuki Sugimoto, Ryuji Yonemitsu, Kozo Matsushita, Satoshi Hisanaga, Masaki Yugami, Yusuke Uehara, Tetsuro Masuda, Takayuki Nakamura, Takuya Tokunaga, Tatsuki Karasugi, Takanao Sueyoshi, Hiro Sato, Yoichiro Iwakura, Kimi Araki, Eisuke Kobayashi, Seiji Okada, Takeshi Miyamoto

**Affiliations:** 1 Department of Orthopedic Surgery, Kumamoto University, 1-1-1 Honjo, Chuo-ku, Kumamoto, 860-8556 Japan; 2 Laboratory of Molecular Cell Biology, School of Pharmaceutical Sciences, Kobe Gakuin University, 1-1-3 Minatojima, Chuo-ku, Koube, 650-8586 Japan; 3 Division of Experimental Animal Immunology, Center for Animal Disease Models, Research Institute for Biomedical Sciences, Tokyo University of Science, 2641 Yamazaki, Noda-Shi, Chiba, 278-8510 Japan; 4 Division of Developmental Genetics, Institute of Resource Development and Analysis, Kumamoto University, 2-2-1 Honjo, Chuo-ku, Kumamoto, 860-0811 Japan; 5 Center for Metabolic Regulation of Healthy Aging, Kumamoto University, 1-1-1, Honjo, Chuo-ku, Kumamoto, 860-8556 Japan; 6 Division of Musculoskeletal Oncology, National Cancer Center Hospital, 5-1-1 Tsukiji, Chuo-ku, Tokyo, 104-0045 Japan; 7 Division of Hematopoiesis, Joint Research Center for Human Retrovirus Infection, Kumamoto University, 2-2-1 Honjo, Chuo-ku, Kumamoto, 860-0811 Japan

**Keywords:** Cancer, Immunology, Oncology

## Abstract

Osteosarcoma is rare but is the most common bone tumor. Diagnostic tools such as magnetic resonance imaging development of chemotherapeutic agents have increased the survival rate in osteosarcoma patients, although 5-year survival has plateaued at 70%. Thus, development of new treatment approaches is needed. Here, we report that IL-17, a proinflammatory cytokine, increases osteosarcoma mortality in a mouse model with AX osteosarcoma cells. AX cell transplantation into wild-type mice resulted in 100% mortality due to ectopic ossification and multi-organ metastasis. However, AX cell transplantation into IL-17-deficient mice significantly prolonged survival relative to controls. CD4-positive cells adjacent to osteosarcoma cells express IL-17, while osteosarcoma cells express the IL-17 receptor IL-17RA. Although AX cells can undergo osteoblast differentiation, as can patient osteosarcoma cells, IL-17 significantly inhibited that differentiation, indicating that IL-17 maintains AX cells in the undifferentiated state seen in malignant tumors. By contrast, IL-17RA-deficient mice transplanted with AX cells showed survival comparable to wild-type mice transplanted with AX cells. Biopsy specimens collected from osteosarcoma patients showed higher expression of IL-17RA compared to IL-17. These findings suggest that IL-17 is essential to maintain osteosarcoma cells in an undifferentiated state and could be a therapeutic target for suppressing tumorigenesis.

## Introduction

Malignant tumors are among the leading causes of death in developed countries^1^. Recently, chronic inflammation has been shown to promote tumorigenesis^2^, and controlling such inflammation, especially in the elderly, is crucial to prevent tumor progression^3^. However, how inflammatory cytokines function in tumor progression in younger individuals has not been fully characterized, since tumorigenesis in that age group is largely promoted by genetic mutations.

Osteosarcoma is the most frequently occurring primary malignant tumor in bone. It is particularly common in children and adolescents^4^ and frequently metastasizes to the lungs and other tissues, leading to death^5^. Osteosarcoma is defined as a tumor derived from mesenchymal stem cells and osteoblastic cells that produce bone-like cells^6^. Ectopic bone formation due to osteosarcoma is frequently detected at both primary tumor sites and metastatic sites^7^. Since similar bone or bone is formed at the final stage of osteoblast differentiation, osteosarcoma can exhibit phenotypes seen in terminally differentiated osteoblastic cells. On the other hand, like other malignancies, osteosarcoma can also exhibit undifferentiated phenotypes, which are considered required for tumor progression. However, how both differentiated and undifferentiated phenotypes are regulated concomitantly in osteosarcoma remains largely unknown.

Osteosarcomas often promote local inflammation, and several inflammatory cytokines are reportedly expressed in sera from osteosarcoma patients, although the role of inflammation in osteosarcoma remains unclear^8^. Recently, use of multi-agent systemic chemotherapy and development of diagnostic imaging techniques such as MRI have improved treatment outcomes, and the 5-year survival rate is now > 70%^9^, although progress in treatments leading to improved outcomes remains limited despite preclinical and clinical studies^10^. Moreover, adjuvant therapy options that could prolong life in cases of unresectable local recurrence or multiple distant metastases after multimodality treatment remain very limited. Clearly, new breakthroughs are needed in treating osteosarcoma, although its infrequent occurrence makes it challenging to develop those regimens.

Previously, a mouse model that recapitulates human osteosarcoma and shows histological bone formation, distant metastasis to multiple organs, and death due to malignancy, was developed^11^. In this model, wild-type mice transplanted subcutaneously or in the abdominal cavity with murine AX cells, which are Ink4a-deficient and overexpress the oncogene c-Myc, showed tumor formation with bone formation at transplant and metastatic sites, as well as 100% mortality^11^. Previously, we observed elevated blood levels of TNFα, an inflammatory cytokine, in AX cell-bearing mice and reported that TNFα knockout significantly suppressed tumor formation and death from malignancy in these mice^12^. However, since tumorigenesis was not completely suppressed in TNFα knockout mice, we hypothesized that other inflammatory cytokines may also promote osteosarcoma progression.

The IL-17 family of inflammatory cytokines is encoded by six genes, IL-17A-F. IL-17A in particular is known to induce other inflammatory cytokines and chemokines and thought to induce inflammation and protection against bacterial infection^13,14^. Also, IL-17A has recently been suggested to have a pro-survival effect on tumor cells in various cancers^15–19^, and several studies of mice transplanted with cancer cells deficient in either IL-17A or its receptor report inhibition of tumor growth^17,20,21^.

Here, we found that IL-17 produced by CD4-positive lymphocytes maintains osteosarcoma cells in an undifferentiated state and is required for tumor progression. IL-17 knockout mice transplanted with AX cells showed markedly decreased tumor growth and significantly prolonged survival compared to similarly transplanted wild-type mice. Accordingly, CRISPR/Cas9 knockout of the IL-17 receptor in AX cells prolonged survival of wild-type mice in vivo. AX cells can differentiate into osteoblastic cells, as can human osteosarcoma cells, but IL-17 stimulation inhibited AX cell osteoblastic differentiation and maintained those cells in an undifferentiated state in vitro. These findings suggest that the inflammatory cytokine IL-17 is required to promote tumor progression even in cells genetically modified to lack Ink4a and overexpress c-Myc. Thus IL-17 represents as a potential therapeutic target to inhibit osteosarcoma progression.

## Results

### IL-17 is required for development of AX osteosarcoma in mice

**Figure 1:**
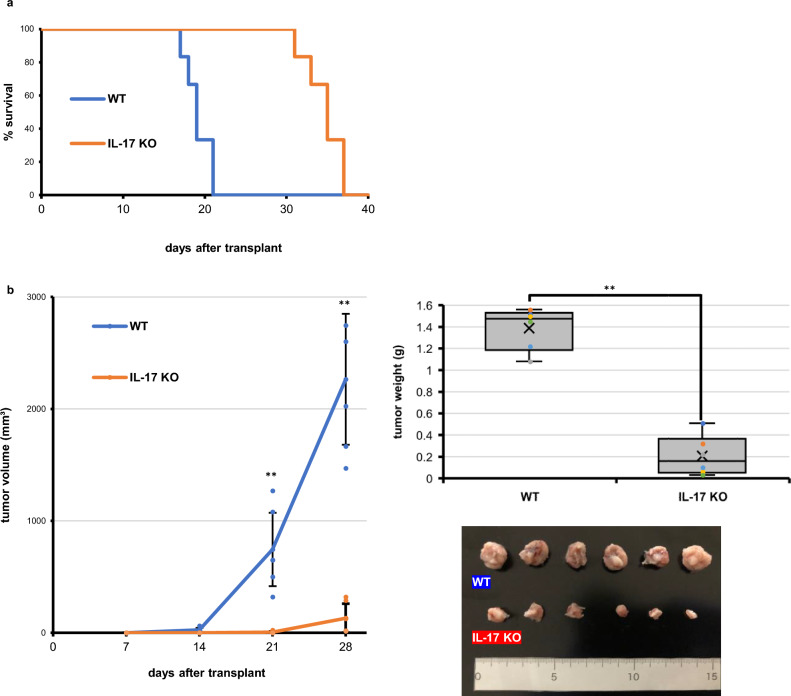
IL-17 signaling is required for osteosarcoma progression in mice. (**a**) IL-17-deficient or wild-type mice were transplanted with AX cells intraperitoneally and survival curves were analyzed (n = 6). (**b**) IL-17-deficient or wild-type mice were transplanted with AX cells subcutaneously, and the volume of resulting tumors was determined on 7, 14, 21, and 28 days after transplantation (left). To do so, major and minor axes of subcutaneous tumors were measured with Vernier Calipers, and tumor volume was calculated using the formula: [tumor volume] = [short diameter^2^ × long diameter]/2. Data represents mean tumor volume (mm^3^) ± SD (each with n = 6, ***P < 0.001). The weight of resulting tumors was also determined on 28 days after transplantation (upper right). Data represents mean tumor weight (g) ± SD (each with n = 6, ***P < 0.001). Shown at lower right are size comparisons of representative IL-17-deficient or wild-type tumors on day 28.

Initially, our goal was to inhibit AX osteosarcoma development in wild-type mice using various agents in vivo. Osteosarcoma arises from osteoblastic cells, which produce osteoid; thus, we first administered rapamycin, which inhibits bone formation^22^, to wild-type mice at the time of AX cell transplant and afterwards. That treatment, however, had no effect on mouse survival relative to untreated controls (Supplementary Fig. S1). Osteoblast bone formation is also reportedly stimulated after osteoclastic bone resorption via growth factor release from bone matrix protein^23–25^. Also men with osteosarcoma reportedly have a poorer prognosis compared to women^26^. However, administration of either zoledronate, a strong inhibitor of osteoclasts, or 17β-estradiol did not prolong survival of AX cell-transplanted wild-type mice (Supplementary Fig. S1). Given that chronic inflammation also functions tumor progression^2^, we also administered anti-inflammatory agents including methotrexate, baricitinib or neutralizing antibody against IL-6 receptor but none enhanced survival of wild-type, AX cell-transplanted mice (Supplementary Fig. S1). Finally, since IL-17 is an inflammatory cytokine, we transplanted AX cells subcutaneously into IL-17A/F-deficient mice (hereafter call IL-17-deficient mice), and found that those mice exhibited significantly prolonged survival compared to similarly transplanted wild-type mice (Fig. 1a). Similar to the subcutaneous injection model, IL-17-deficient mice transplanted in the bone marrow cavity with AX cells exhibited significantly prolonged survival relative to similarly transplanted wild-type mice (Supplementary Fig. S2). Thus, we chose IL-17-deficient mice for further analysis.

We next measured the size of subcutaneous tumors in IL-17-deficient and wild-type mice at 7, 14, 21, and 28 days after AX cell transplantation into the lateral abdomen. Tumors were significantly smaller in IL-17-deficient relative to wild-type mice (Fig. 1b), suggesting that IL-17 stimulation is required for in AX cell tumor progression in vivo.

### IL-17 is produced by bone marrow-derived CD4 + cells in wild-type mice transplanted with AX cells

**Figure 2:**
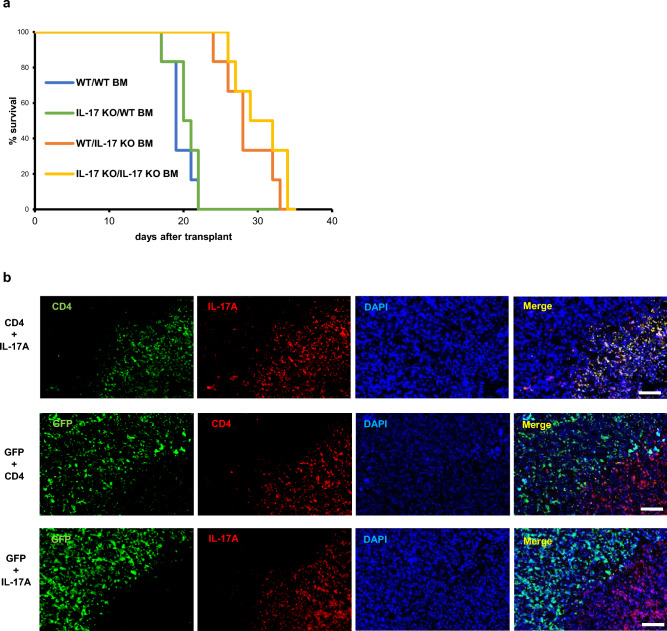
IL-17 is produced by bone marrow-derived CD4 + lymphocytes. (**a**) IL-17 knockout (IL-17 KO) or wild-type (WT) mice were lethally-irradiated, and subsequently BM of each line was reconstituted by transplantation of BM cells from either wild-type or IL-17 knockout mice to establish BM chimeric mice. Two months later, mice were transplanted with AX cells intraperitoneally, and survival curves were analyzed (n = 6). (**b**) Wild-type mice were transplanted subcutaneously with AX cells, which harbor a GFP marker, and a month later, frozen tumor sections were prepared. Frozen sections were stained with rat anti-CD4, rabbit anti IL-17A or chicken anti-GFP antibody followed by Alexa488 or Alexa594-conjugated anti-rat Ig’, Alexa594-conjugated anti-rabbit Ig’ or Alexa488-conjugated anti-chicken Ig’ antibody, respectively, and observed under a fluorescent microscopy (right). DAPI served as a nuclear marker. Bar, 100 μm.

Next, to establish bone marrow (BM) chimeric mice, we transplanted both lethally-irradiated wild-type and IL-17-deficient mice each with either BM cells from wild-type or IL-17-deficient mice (Fig. 2a). We then transplanted AX cells, which harbor a GFP marker, into each of these lines and assessed animal survival. Survival of wild-type mice reconstituted with IL-17-deficient BM cells was significantly greater than that of IL-17-deficient mice transplanted with wild-type BM cells (Fig. 2a), suggesting that IL-17 is produced by BM cells. Immunochemical analysis of tumors from these mice showed localization of IL-17A-expressing cells near GFP-positive AX cells (Fig. 2b). Moreover, IL-17A expressing cells were also CD4-positive in wild-type mice, whereas in IL-17-deficient mice, we did not detect CD4/IL-17A double-positive cells, although CD4-positive cells accumulated in tumor regions as they do in wild-type mice (Fig. 2b and Supplementary Fig. S3). These results strongly suggest that immune cells in the tumor microenvironment produce IL-17.

### AX cells express IL-17RA

**Figure 3:**
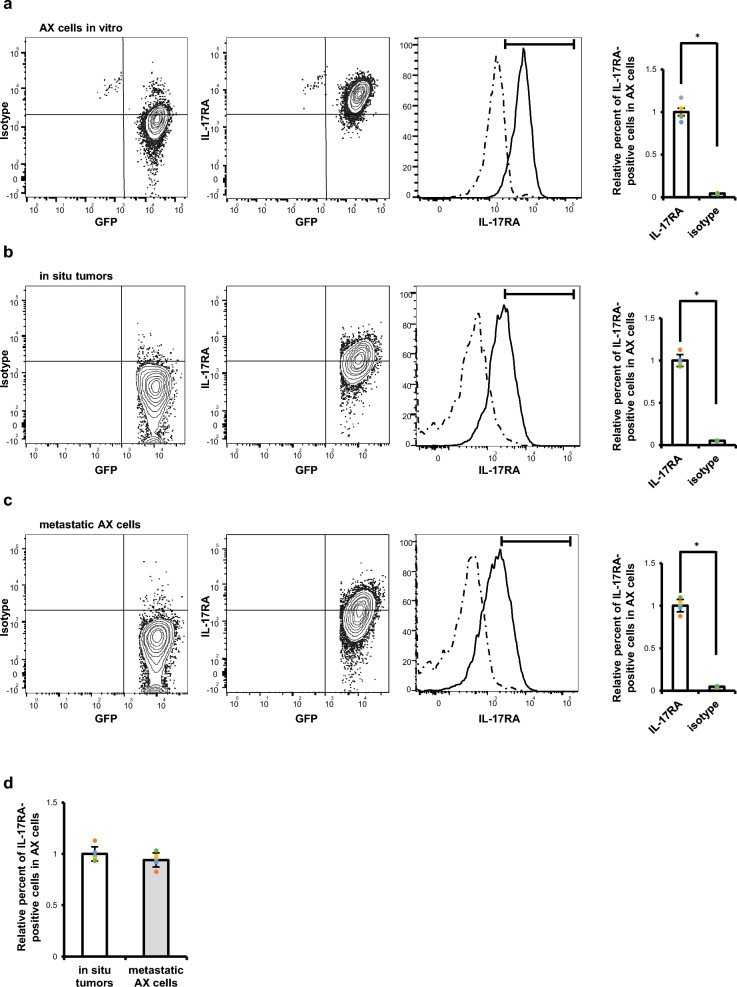
IL-17RA is expressed on the AX cell surface. (**a**) IL-17RA expression on cultured AX cells, which harbor a GFP tag, was analyzed by flow cytometry (n = 5). (**b**,**c**) Wild-type mice were transplanted with AX cells subcutaneously and a month later, tumors were harvested from transplanted subcutaneous (**b**) or metastatic lung sites (**c**), and IL-17RA expression was analyzed by flow cytometry (n = 5). Solid or dotted lines in histograms represent AX cells stained with anti-IL-17RA or isotype control antibody, respectively. Graph shows mean IL-17RA expression relative to isotype control ± s.d (n = 5, *P < 0.05). (**d**) Graph shows mean IL-17RA expression levels in metastatic relative to in situ AX cells ± s.d (n = 5, *P < 0.05).

IL-17 signals are transduced through IL-17 receptor A (IL-17RA) and C (IL-17RC) complexes, and our flow cytometric analysis revealed IL-17RA expression on cultured AX cells (Fig. 3a). One month after subcutaneous transplantation of AX cells into wild-type mice, we harvested tumors developed from transplanted subcutaneous (Fig. 3b) or lung metastasized (Fig. 3c) sites and detected IL-17RA in AX cells harvested from transplanted mice by flow cytometry (Fig. 3b–d). IL-17RA expression levels were equivalent in AX cells from metastatic and in situ tumors (Fig. 3d). Thus, IL-17RA expression on AX cells remains as tumors develop.

### IL-17 inhibits osteoblastic differentiation of AX cells

**Figure 4:**
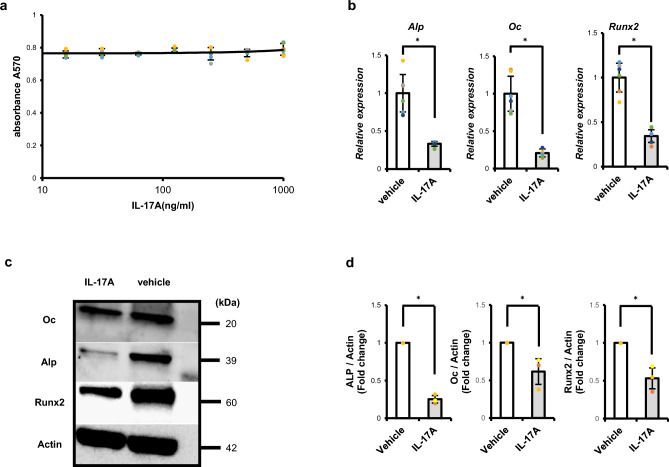
IL-17 signaling inhibits osteoblastic differentiation of AX cells. (**a**) AX cells were cultured 24 h with indicated concentrations of IL-17A (10–1000 ng/ml) and the number of AX cells analyzed by MTT [3-(4,5-dimethylthiazol-2-yl)-2,5-diphenyltetrazolium bromide] assay based on absorbance measuring at 570 nm. Data represent mean absorbance ± s.d. (n = 6, *P < 0.05). (**b**) AX cells were cultured 24 h in the presence (IL-17A) or absence (vehicle) of IL-17A (500 ng/ml), total RNA was prepared, and expression of *ALP*, *Osteocalcin* (*Oc*) or *Runx2* relative to *β-actin* was analyzed by quantitative real-time PCR. Data represent mean expression relative to *β-actin* ± s.d. (n = 6, *P < 0.05). (**c**) AX cells were cultured 48 h in the presence (IL-17A) or absence (vehicle) of IL-17A (500 ng/ml), total protein was extracted, and ALP, Oc and Runx2 levels were analyzed by western blot. (**d**) The graph shows the normalization of ALP, Oc and Runx2 levels to total Actin levels (*P < 0.05, n = 3).

We next analyzed effects of IL-17 signaling in AX cells in vitro. Following IL-17A treatment for 24 h, AX cells did not exhibit increased AX cell proliferation in vitro (Fig. 4a) Interestingly, however, expression of osteoblastic genes, such as *alkaline phosphatase* (*ALP*), *osteocalcin* (*Oc*) and *Runx2*, was significantly downregulated in AX cells following IL-17A treatment in vitro (Fig. 4b), in addition, changes at the protein level were also align with gene expression levels (Fig. 4c,d), suggesting that IL-17 signaling inhibits osteoblastic differentiation in this context and maintains osteosarcoma cells in an undifferentiated state. As in AX cells, proliferation of MG63 cells, a human osteosarcoma line, was not stimulated by IL-17A (Supplementary Fig. S4). Moreover, although *Oc* expression was equivalent, expression of *ALP* and *Runx2* was significantly inhibited in MG63 cells by IL-17A stimulation in vitro, as it was in AX cells (Supplementary Fig. S4).

**Figure 5:**
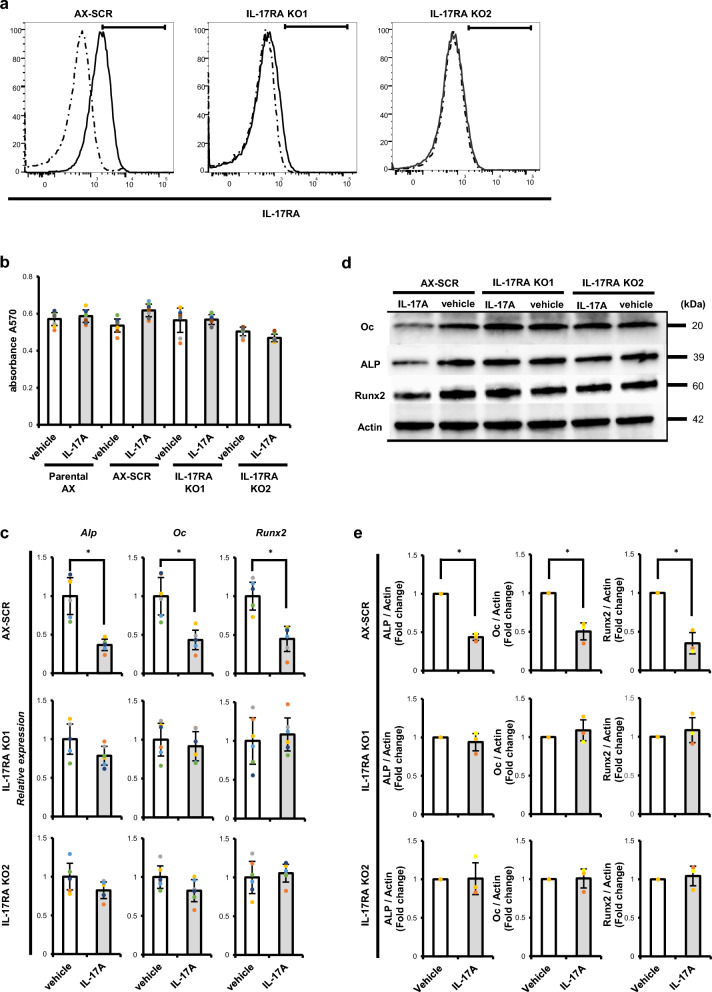
Establishment of IL-17RA-deficient AX cells. (**a**) Two independent IL-17RA-deficient AX lines (KO1 and KO2) were generated by lentiviral transduction using the CRISPR/Cas9 system. AX cells transduced with scramble constructs served as controls (AX-SCR). IL-17RA expression on these AX cell lines, which harbor a GFP tag, was analyzed by flow cytometry. Solid or dotted lines in histograms represent AX cells stained with anti-IL-17RA or isotype control antibody, respectively. (**b**) IL-17RA-deficient and control AX cells (Parental AX cells and AX-SCR) were cultured 24 h with or without (vehicle) IL-17A (500 ng/ml) and the number of AX cells was analyzed by MTT [3-(4,5-dimethylthiazol-2-yl)-2,5-diphenyltetrazolium bromide] assay measuring absorbance at 570 nm. Data represent mean absorbance ± s.d. (n = 6, *P < 0.05). (**c**) IL-17RA knockout (middle and bottom rows) or scramble-transduced AX cells (top row) were treated with or without (vehicle) IL-17A (500 ng/ml). After 24 h, total RNA was prepared, and expression of *ALP*, *Oc* or *Runx2* relative to *β-actin* was analyzed by quantitative real-time PCR. Data represent mean indicated gene expression relative to *β-actin* ± s.d. (n = 6, *P < 0.05). (**d**) IL-17RA knockout (middle and right rows) or scramble-transduced AX cells (left rows) were treated with or without (vehicle) IL-17A (500 ng/ml). After 48 h, total protein was extracted, and ALP, Oc and Runx2 levels were analyzed by western blot. The graph shows the normalization of ALP, Oc and Runx2 levels to total Actin levels (*P < 0.05, n = 3).

We then prepared AX cells lacking the IL-17A receptor (IL-17RA) using the CRISPR/Cas9 system. Control AX cells were transduced with scramble constructs, and IL-17RA knockout was confirmed by flow cytometry (Fig. 5a). Proliferation was comparable in the parental line, 2 independent lines of IL-17RA-deficient cells (IL-17RA KO1 and KO2) and control SCR-AX cells, and was unchanged in any line by IL-17A treatment in vitro (Fig. 5b).

**Figure 6:**
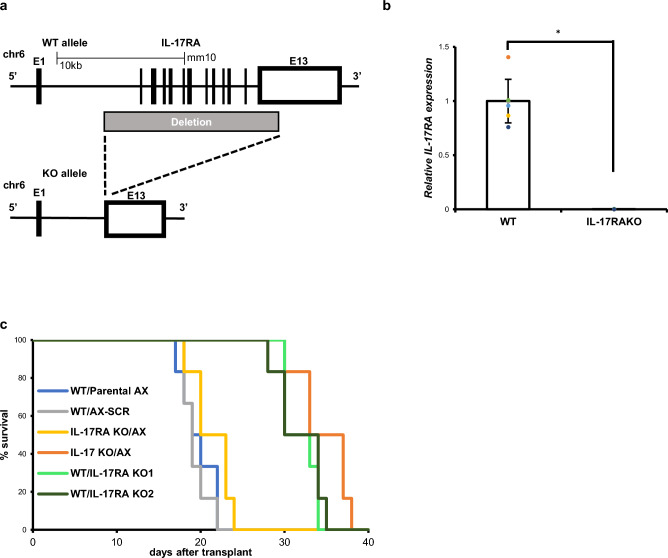
IL-17RA on AX cells is required for osteosarcoma progression. (**a**) Structure of the mouse *IL-17RA* gene locus (WT allele), and the predicted mutant *IL-17RA* gene (KO allele). (**b**) Splenocytes were isolated from wild-type or IL-17RA-deficient mice, and *IL-17RA* expression was analyzed by real-time PCR. Data represent mean *IL-17RA* expression relative to *β-actin* ± s.d. (n = 6, *P < 0.05). (**c**) Survival of mice with IL-17 ligand knockout (IL-17 KO), IL-17RA receptor knockout (IL-17RA KO) or wild-type (WT) mice, transplanted with AX cells of indicated genotypes (parental, IL-17RA KO1, IL-17RA KO2 or SCR) (n = 6).

Next, we examined expression of osteoblastic genes and proteins in vitro in IL-17RA-deficient versus SCR-AX cells in the presence or absence of IL-17A treatment. We found that inhibition of osteoblastic differentiation by IL-17A seen in SCR-AX cells was not evident in either line of IL-17RA-deficient AX cells (KO1 or KO2) (Fig. 5c–e). These results were confirmed by Alizarin Red S staining, which detected mineralized matrix production in both lines (Supplementary Fig. S5). Mineralized matrix production following treatment with osteogenic medium was significantly inhibited in parental and SCR-AX cells, but that inhibition was blocked in IL-17RA-deficient AX cells (KO1 or KO2) (Supplementary Fig. S5). However, analysis of wild-type mice transplanted with IL-17RA-deficient (KO1 or KO2), control (SCR-AX) or parental AX (AX) cells indicated that wild-type mice transplanted with IL-17RA-deficient AX cells survived for a significantly longer period of time than did wild-type mice transplanted with control or parental AX cells (Fig. 6c).

Finally, to confirm that IL-17 signaling in tumor rather than host cells is required for tumor progression, we established mice globally deficient in IL-17RA (Fig. 6a,b). Loss of IL-17RA expression in IL-17RA-deficient mice was confirmed by real-time PCR and flow cytometric analysis of splenocytes (Fig. 6b and Supplementary Fig. S6). Survival of those IL-17RA-deficient mice following transplant with parental AX cells was equivalent to that of similarly-transplanted wild-type mice (Fig. 6c). Microarray analysis revealed that osteosarcoma samples from patients exhibited a higher IL-17RA expression relative to either IL-17A and IL-17F (Supplementary Fig. S7). Taken together, our data indicate that either IL-17 or IL-17RA represents as a therapeutic target to inhibit osteosarcoma progression and likely to improve survival rate of the osteosarcoma patients.

## Discussion

Osteosarcoma is a rare malignant tumor that occurs in growing teenagers and the elderly, and is characterized by both undifferentiated phenotypes that result in malignant tumors and the ability to differentiate into osteoblast-like cells that produce osteoid/bone, a terminally differentiated osteoblastic phenotype concomitantly. However, it is not clear how an undifferentiated state is maintained or how osteosarcoma tumor development is regulated. Our results indicate that IL-17, an inflammatory cytokine, may maintain that undifferentiated state and be required for osteosarcoma progression in an AX model.

Various factors including stressors, environmental pollutants, dietary factors, and bacterial or viral infections, all of which can induce inflammation, can promote cancer development and progression^27^. In addition, serum cytokine levels in patients with breast or colorectal cancer are reportedly higher than in healthy individuals^28^. In osteosarcoma patients, local inflammation is frequently detected^8^, and other sarcomas, such as Ewing’s sarcoma, also exhibit inflammation^29^. Thus, inflammatory cytokines may play a key role in sarcoma tumorigenesis and progression.

Gene mutations frequently function in tumor progression^30^. By contrast, chronic inflammation is known to promote tumorigenesis in the elderly^3^. AX osteosarcoma cells are INK4a-deficient and overexpress the oncogene c-Myc, both features of osteosarcoma seen in humans^11^. It was unanticipated that malignant tumors induced by gene mutation required activity of the inflammatory cytokine IL-17 for tumorigenesis. FGF2 produced by tumor-associated fibroblasts reportedly contributes to maintenance of tumor cell immaturity and aggressiveness^31^. Our results indicate that the IL-17/IL-I7RA axis acts either to suppress AX cell differentiation or promote tumorigenesis. Since chemotherapeutic agents generally target the cell cycle, combining them with therapies that target IL-17, or potentially its receptor, may have stronger antitumor effects than conventional treatments.

Osteosarcomas can differentiate into cells exhibiting a terminally differentiated osteoblast phenotype, given that tumors produce osteoid and bone at the final stage of osteoblastic differentiation. Osteosarcoma cells reportedly produce the osteogenic factor bone morphogenic protein 2 (BMP2) and form ectopic bone^32,33^. Moreover, they remain proliferative in an undifferentiated state, which is terminated in normal cells by differentiation signals. In malignant tumors, a block in normal differentiation promotes tumor progression and allows continuous tumor growth. Therapies inducing of tumor cell differentiation have been investigated for numerous malignancies including acute promyelocytic leukemia (PML)^34^. Our findings indicate that differentiation-inducing therapies may be applicable to osteosarcoma therapies.

Here, we showed that the IL-17 receptor, IL-17RA, is expressed in AX cells and that IL-17 activates those receptors. The human osteosarcoma cell lines U-2, MG-63 and HOS reportedly express the IL-17 receptor^35^. Here, we also used a microarray of 33 human osteosarcoma samples to confirm IL-17RA expression in osteosarcoma isolated from patients (Supplementary Fig. S7). These results further support the idea that IL-17 or the IL-17A receptor is a potential therapeutic target for human osteosarcoma.

While an association of IL-17 with various malignancies has been reported^15–21^, mechanisms underlying tumor progression driven by IL-17 are not clear. Some reports suggest that IL-17 promotes tumor growth by inducing IL-6 production to activate IL-6-Stat3 signaling^36^. Others report that IL-17 loss reduces the number of natural killer and T cells, which produce interferon, and thus enable tumor growth^37^. Relevant to osteosarcoma, serum IL-17A levels are reportedly elevated in patients with metastatic osteosarcoma, and IL-17A/IL-17RA interaction reportedly promotes osteosarcoma metastasis in nude mice^38^. Here, we show that IL-17 likely produced from CD4-positive T cells acts directly on osteosarcoma cells to promote tumor growth by inhibiting osteoblastic differentiation. This finding may also be relevant to pathogenesis of tumor progression in sarcomas.

An association of IL-17 with osteoblasts and osteoclasts was described previously^39^. In rheumatoid arthritis (RA), IL-17-producing Th17 cells reportedly induce local inflammation by stimulating production of IL-6, IL-1, TNFα and chemokines, and IL-17 may directly stimulate synovial fibroblasts to induce RANKL expression, which in turn, results in bone destruction^39^. Ankylosing spondylitis (AS) is a disease characterized by inflammation at the enthesis of tendons, leading to spinal ankylosis with ectopic ossification of spines. IL-17-producing T cells are also detected at enthesopathy sites in AS patients^40,41^, and neutralizing IL-17 antibody effectively inhibits disease in AS patients and has been approved clinically to treat those patients^42^. Furthermore, IL-17-producing γδT cells promote fracture healing by stimulating osteogenesis via osteoblasts during fracture healing in a mouse fracture model^43^. Thus, IL-17 has dual activating and inhibitory effects on osteoblasts. Like IL-17, TNFα also reportedly exhibits such dual effects on osteoblasts^12,44^. Nonetheless, our study and other reports indicate that the IL-17-IL-17RA axis represents as a therapeutic option to inhibit bone-related diseases such as osteosarcoma, RA and AS.

## Materials and methods

### Ethical conduct of research

The authors declare that all protocols used to obtain and process all human samples were approved by Kumamoto University Certified Clinical Research Review Board and National Cancer Center Hospital Research Ethics Review Committee. All research was performed in accordance with the Hospital Ethical Guidelines of Kumamoto University Hospital and the National Cancer Center hospital. All research was conformed to the ethical guidelines of the Helsinki Declaration. Donors provided written informed consent before obtaining samples.

### Chemicals and reagents

Baricitinib was purchased from Cayman Chemical Company (Michigan, USA). 17β-Estradiol and Methotrexate were purchased from Nacalai Tesque (Kyoto, Japan). Neutralizing antibody against the IL-6 receptor was provided by Chugai Pharmaceutical Co., Ltd (Tokyo, Japan). Zoredonic acid hydrate (Zometa) was purchased from Novartis (Basel, Switzerland). Rapamycin was purchased from LC Laboratories (Woburn, MA, USA). Mouse IL-17A was purchased from PeproTech Ltd (London, UK). Collagenase Type1 was purchased from Thermo Fisher Scientific K.K (Tokyo, Japan).

### Animal studies

Wild-type mice were purchased from Japan SLC, Inc. (Sizuoka, Japan). IL-17-deficient mice were established previously^14^.

IL-17RA knockout (KO) mice were generated by electroporation of C57BL/6 J fertilized eggs. The electroporation solution contained 10 μM tracrRNA (GE-002; Fasmac), 10 μM synthetic crRNA (Fasmac), 0.1 μg/μl Cas9 protein (317-08441; Nippon Gene), and 1 μg/μl ssODN in Opti-MEM I Reduced Serum Medium (31985062; Thermo Fisher Scientific). Synthetic crRNAs were designed to recognize the sequence GATGTTTCCTCTATTACCCG (GGG) in the IL-17RA 1st intron and GTGGATCTGTTGCCCTACGG (GGG) in the 3′UTR. ssODN (5′-ACAATATACACCCTCTTAAGTAGCAGGCTCAGGAGGCCTACAGGTCCTCTTCAGAGATCAGCTTCTGCTCAGAGCCGGAGGGATTCTGCTCTTCTGGGGTGGGTCTCCGTGGCTCC-3′) served as a homologous recombination template. Electroporation was carried out using Super Electroporator NEPA 21 (NEPA GENE) on Glass Microslides and round wire electrodes, with a 1.0 mm gap (45–0104; BTX). Four steps of square pulses were applied: (1) three 3 mS poring pulses at 97 mS intervals at 30 V; (2) three 3 mS polarity-changed poring pulses at 97 mS intervals at 30 V; (3) five 50 mS transfer pulses at 50 mS intervals at 4 V with 40% decay of voltage per pulse; and (4) five 50 mS polarity-changed transfer pulses at 50 mS intervals at 4 V with 40% decay of voltage per pulse. Embryos were subsequently cultured in KSOM medium overnight and then transferred into oviducts of foster mothers at the two-cell stage.

All animals were maintained under specific pathogen-free conditions in animal facilities certified by the Kumamoto University School of Medicine Animal Care Committee. All experiments were performed according to the guidelines approved by the Institutional Animal Experiment Committee of Kumamoto University School of Medicine (A2022-019). The study was carried out in compliance with the ARRIVE guidelines. Euthanasia was performed by isoflurane anesthesia followed by cervical dislocation according to the 2013 AVMA guidelines. All animal use was conducted in accordance with the Animal Welfare Act. We injected a total of 2.5 × 10^6^ AX cells into 8-week-old female mice intraperitoneally, subcutaneously or into the bone marrow cavity and assessed survival curves. Size of subcutaneously transplanted tumors was analyzed at 7, 14, 21, and 28 days after transplantation.

In this study, a humanitarian endpoint was set as below, and mice meeting any of these criteria were euthanized.

1. Tumor weight exceeding 10% of body weight, or tumor diameter exceeding 20 mm.
2. When ulceration, necrosis, or infection of the tumor was observed.
3. When there was a disturbance in water intake or feeding.
4. Weight loss of 20% or more relative wild-type mice of the same weeks-of-age, or weight loss of 25% or more in a 7-day period.

### Cell culture and real-time PCR analysis

AX cells were established previously^11^ and maintained in DMEM (Sigma-Aldrich, St Louis, MO, USA) containing 10% FBS, 1% GlutaMax and antibiotics. Human osteosarcoma MG63 cells were maintained in DMEM (Sigma-Aldrich, St Louis, MO, USA) containing 10% FBS, and antibiotics. AX or human osteosarcoma MG63 cells were cultured in the presence or absence of 500 ng/ml mouse or human recombinant IL-17A, respectively, for 24 h as previously described^43^. Splenocytes were isolated from wild-type or IL-17RA-deficient mice. Total RNAs were isolated from cultured cells or from splenocytes using TRIzol reagent (Invitrogen, Tokyo, Japan). cDNAs were synthesized from total RNAs using PrimeScript RT Master Mix (Takara Bio Inc.). Real-time PCR was performed using TB Green Premix ExTaq II (Takara Bio Inc.) with a DICE Thermal cycler (Takara Bio Inc.), according to the manufacturer’s instructions. Samples were matched to a standard curve generated by amplifying serially diluted products using the same PCR reactions. *β-actin* served as an internal control. Primer sequences used for analysis in AX (mouse) or MG63 (human) cells were as follows:

Mouse primers.

*β-actin* forward: 5′-TGAGAGGGAAATCGTGCGTGAC-3′;

*β-actin* reverse: 5′-AAGAAGGAAGGCTGGAAAAGAG-3′;

*Alkaline phosphatase* (*ALP*) forward: 5′-ACACCTTGACTGTGGTTACTGCTGA-3′;

*Alkaline phosphatase* (*ALP*) reverse: 5′-CCTTGTAGCCAGGCCCGTTA-3′;

*Osteocalcin* (*Oc*) forward: 5′-TAGCAGACACCATGAGGACCCT-3′;

*Osteocalcin* (*Oc*) reverse: 5′-TGGACATGAAGGCTTTGTCAGA-3′;

*Runx2* forward: 5′-GACGTGCCCAGGCGTATTTC-3′;

*Runx2* reverse: 5′-AAGGTGGCTGGGTAGTGCATTC-3′;

*IL-17RA* forward: 5′-AGTGTTTCCTCTACCCAGCAC-3′;

*IL-17RA* reverse: 5′-GAAAACCGCCACCGCTTAC-3′.

Human primers.

*β-actin* forward: 5′-GCCCTGAGGCACTCTTCCA-3′;

*β-actin* reverse: 5′-CGGATGTCCACGTCACACTTC-3′;

*Alkaline phosphatase (ALP)* forward: 5′-ACCATTCCCACGTCTTCACATTT-3′;

*Alkaline phosphatase (ALP)* reverse: 5′-AGACATTCTCTCGTTCACCGCC-3′;

*Osteocalcin (Oc)* forward: 5′-AGGTGCAGCCTTTGTGTCCA-3′;

*Osteocalcin (Oc)* reverse: 5′-GGCTCCCAGCCATTGATACAG-3′;

*Runx2* forward: 5′-CGGAATGCCTCTGCTGTTAT-3′;

*Runx2* reverse: 5′-AGCTTCTGTCTGTGCCTTCT-3′.

### Cell count assay

The number of AX and human osteosarcoma MG63 cells was determined using a MTT Assay Kit (BioAssay Systems, (Hayward, CA, USA). Cells (1.0 × 10^4^ viable cells/ml) were seeded into 96-well plates and left to adhere overnight. Cells were then incubated 24 h in a humidified 5% CO_2_ incubator at 37 °C in the presence or absence of indicated IL-17A concentrations. Subsequently, a MTT (0.5 mg/ml final conc.) solution was added to each well. After a 3 h incubation, 100 μl of acidified isopropanol (HCl 34 μl/10 ml isopropanol) was added to dissolve crystals. Absorption values at 570 nm were determined using an iMark Microplate Absorbance Reader (BIO-RAD, Hercules, CA, USA). Values are normalized to untreated (Control) samples.

### Generation of IL-17rRA knockout (KO) AX cells

IL-17RA KO AX lines were generated using a lentiviral CRISPR/Cas9 system. The lentiviral pLV[2CRISPR]-hCas9:T2A:Puro-U6 expression vector (VectorBuilder, Inc., Chicago, IL, USA) containing the sequence of murine IL-17RA gRNAs (two independent sequences) or scramble gRNA were used. gRNA sequences were as follows.

IL-17RA-1:

CTGAAGGAAAACCGCCACTG

GACCTGGAGATGTTTGAACC

IL-17RA-2:

GCTGTCATCTTACCCGCTTA

AACAACGTAGGTGCCGAAGC

Lenti-X™ 293 T cells (Nacalai Tesque, Kyoto, Japan) were transfected with each vector plus the envelope plasmid pMD2.G and packaging plasmid psPAX2. Following 48 h of incubation, supernatants were collected and filtered through a 0.45-μm low-protein binding Durapore membrane. AX cells were transduced 24 h with the lentiviral vector, followed by a complete medium change. Forty-eight hour post-transfection, medium was changed to puromycin-containing medium (2000 ng/ml), and puromycin-resistant cells were selected for 7 days. To confirm allelic knockouts, single-cell colonies were isolated, and IL-17RA knockout was confirmed by flow cytometry.

### Flow cytometry

AX cells cultured on tissue culture plates were collected and transplanted subcutaneously into wild-type mice. One month later, tumors were harvested from transplanted subcutaneous sites and metastatic lung sites and treated with collagenase 1 at 37 °C for 6 h to isolate AX cells, which were stained with APC-conjugated rat anti-mouse IL-17RA or APC-conjugated rat IgG2a isotype control antibody (Thermo Fisher Scientific K.K, Tokyo, Japan). For flow cytometry analysis, splenocytes were isolated from wild-type or IL-17RA-deficient mice and stained with APC-conjugated rat anti-mouse IL-17RA or APC-conjugated rat IgG2a isotype control antibody (Thermo Fisher Scientific K.K, Tokyo, Japan). Cells were stained with PI at a concentration of 1.0 μg/ml in PBS before LSR II analysis. Flow cytometry data was analyzed using FlowJo software ver.10.

### Histopathology and fluorescent immunohistochemistry

Wild-type mice were transplanted with AX cells subcutaneously and a month later, tumors were harvested, fixed with 4% PFA/PBS, and frozen in SCEM-L1 compound (Section-lab, Hiroshima, Japan). For fluorescent immunohistochemistry, 5 μm thick sections were cut and treated 15 min with 0.05% Proteinase K/PBS for antigen retrieval. After blocking 30 min at room temperature with 3% BSA/PBS, sections were incubated 12 h at 4℃ with rabbit anti-mouse IL-17A (abcam, Cambridge, UK, diluted 1:100), chicken anti-GFP (abcam, Cambridge, UK, diluted 1:100) or rat anti-mouse CD4 (R&D systems, Inc., Minneapolis, MN, USA, diluted 1:100).

After washing with PBS, sections were incubated with Alexa Fluor 594-conjugated Goat anti-rat IgG, Alexa Fluor 594-conjugated Goat anti-rabbit IgG, Alexa Fluor 488-conjugated Goat anti-chicken IgG or Goat anti-rat IgG (Invitrogen) antibody (each diluted 1:200). Sections were then mounted in Vectashield mounting medium for fluorescence H-1200 (Vector Laboratories, Inc., Newark, CA, USA). Nuclei were stained with DAPI. Images were acquired with a fluorescence microscope (BZ800, KEYENCE, Osaka, Japan).

### Western blot

Western blotting was performed using a Bio-Rad Criterion™ Cell electrophoresis system. Cultured AX cells were homogenized at 4 °C in 300 μL of lysate (Tissue Protein Extraction Reagent [Thermo Fisher Scientific K.K, Tokyo, Japan])-containing protease inhibitor (cOmplete ULTRA Tablets, Mini, EASYpack [Sigma-Aldrich, Tokyo, Japan]). Lysates were centrifuged 15 min at 15,000 rpm to extract proteins.

Proteins were resolved on 10% polyacrylamide gels and transferred to polyvinylidene difluoride (PVDF) membranes, which were blocked 30 min with 5% skimmed milk. Membranes were then incubated 1 h with primary antibodies, namely, rabbit anti-mouse ALP (Abcam, Cambridge, UK, diluted 1:1000), rabbit anti-mouse Oc (Abcam, Cambridge, UK, diluted 1:1000), rabbit anti-mouse Runx2 (cell signaling technology, MA, USA, diluted 1:1000) and rabbit anti-Actin (Abcam, Cambridge, UK, diluted 1:1000), at room temperature and then 1 h with Goat anti-rabbit IgG conjugated to horseradish peroxidase (Abcam, Cambridge, UK, diluted 1:2000) at room temperature. Signals were detected by chemiluminescence using the BioRad Clarity Western ECL substrate and visualized using a ChemiDoc™ MP Imaging System. Bands intensities were normalized to that of Actin using the Image Lab software and reported as arbitrary densitometry units.

### Alizarin Red S staining and measurement of mineralization

AX and IL-17RA KO AX cells were resuspended in DMEM containing 10% FBS and seeded in a culture dish. After a 2-day incubation, cells were resuspended in osteogenic medium (Osteoblast-Inducer Reagent, Takara Bio Inc.) and seeded in 6-well plates (2.0 × 10^4^ cells per well). Culture medium was changed every 2 days. Recombinant mouse IL-17A (Peprotech Ltd) was added to a final concentration of 500 ng/ml at the time of induction of osteoblastogenesis when the media was changed. Alizarin Red S staining and evaluation of mineralization were performed on day 14. After washing cells three times with PBS, 100% methanol at − 20 degrees was added to fix cells. Then, after washing with dH_2_O, staining was performed with Alizarin Red S staining solution (pH6.3). Staining solution was washed away with dH_2_O, and stained cells were air-dried. To quantitate mineralization, Alizarin Red S was extracted in 10% acetic acid and assessed for absorbance at 405 nm using an iMark Microplate Absorbance Reader (BIO-RAD). Values are normalized to untreated (Control) samples.

### Statistical analysis

Statistical analysis was performed using Student’s t-test (2 subgroups) or one-way ANOVA (3 or more subgroups), followed by a Tukey–Kramer test to determine significance between groups. In this context, significant differences were defined as *P* < 0.05. Difference in OS was calculated with Kaplan–Meier method, log-rank test. The Bonferroni correction was used to correct for multiple testing when the *P* value needed correction.

### Supplementary Information

Supplementary Figures.

## Data Availability

All data generated or analyzed during this study are included in this published article (and its Supplementary Information files).
