# Supplementary material for: The IL-17-IL-17RA axis is required to promote osteosarcoma progression in mice
Source: Sci Rep. 2023 Dec 7;13:21572. doi: 10.1038/s41598-023-49016-1 (PMC10703823; doi:10.1038/s41598-023-49016-1)

The IL-17-IL-17RA axis is required to promote osteosarcoma progression in mice

Naoto Yoshimura[1], Ryusho Kariya[2,7], Masaki Shimada[1], Makoto Tateyama[1], Hideto Matsunaga[1], Yuto Shibata[1], Shuntaro Tanimura[1], Kosei Takata[1], Takahiro Arima[1], Junki Kawakami[1], Kazuya Maeda[1], Yuko Fukuma[1], Masaru Uragami[1], Katsumasa Ideo[1], Kazuki Sugimoto[1], Ryuji Yonemitsu[1], Kozo Matsushita[1], Satoshi Hisanaga[1], Masaki Yugami[1], Yusuke Uehara[1], Tetsuro Masuda[1], Takayuki Nakamura[1], Takuya Tokunaga[1], Tatsuki Karasugi[1], Takanao Sueyoshi[1], Hiro Sato[1], Yoichiro Iwakura[3], Kimi Araki[4,5], Eisuke Kobayashi[6], Seiji Okada[7] and Takeshi Miyamoto[1]

[1]Department of Orthopedic Surgery, Kumamoto University, 1-1-1 Honjo, Chuo-ku, Kumamoto 860-8556, Japan.
[2]Laboratory of Molecular Cell Biology, School of Pharmaceutical Sciences, Kobe Gakuin University, 1-1-3 Minatojima, Chuo-ku, Koube 650-8586, Japan.
[3]Division of Experimental Animal Immunology, Center for Animal Disease Models, Research Institute for Biomedical Sciences, Tokyo University of Science, 2641 Yamazaki, Noda-shi, Chiba 278-8510, Japan.
[4]Division of Developmental Genetics, Institute of Resource Development and Analysis, Kumamoto University, 2-2-1 Honjo, Chuo-ku, Kumamoto, 860-0811, Japan.
[5]Center for Metabolic Regulation of Healthy Aging, Kumamoto University, 1-1-1, Honjo, Chuo-ku, Kumamoto, 860-8556 Japan.
[6]Division of Musculoskeletal Oncology, National Cancer Center Hospital, 5-1-1 Tsukiji, Chuo-ku, Tokyo 104-0045, Japan.
[7]Division of Hematopoiesis, Joint Research Center for Human Retrovirus Infection, Kumamoto University, 2-2-1 Honjo, Chuo-ku, Kumamoto 860-0811, Japan.

Correspondence should be addressed to: T. Miyamoto, Department of Orthopedic Surgery, Faculty of Life Sciences, Kumamoto University, 1-1- Honjo, Chuo-ku, Kumamoto 860-8556, Japan
TEL: 81-96-373-5226, FAX: 81-96-373-5228, e-mail: miyamoto.takeshi@kuh.kumamoto-u.ac.jp

Supplemental Figure Legends

Supplemental Figure S1. Effects of various agents on survival of wild-type mice transplanted with AX cells.

(a-f) Wild-type mice were transplanted with AX cells intraperitoneally, treated with or without indicated reagents as described below, and analyzed for survival (n=6). Injections of vehicle served as controls. (a) Rapamycin (120 μg/100 μl 10% DMSO-PBS) was administered subcutaneously three times a week. (b) Zoledronate (10 μg/100 μl PBS) was administered subcutaneously once a week. (c) 17β-estradiol (1mg/200μl 10% DMSO-PBS) was administered intraperitoneally 5 times a week. (d) Methotrexate (500 μg/200 μl 10% DMSO-PBS) was administered intraperitoneally twice a week. (e) Baricitinib (300 μg/200 μl 10%DMSO-PBS) was administered intraperitoneally 6 times a week. (f) IL-6R antibody (500 μg/100 μl PBS) was administered intraperitoneally once a week.

Supplemental Figure S2. Survival curves of wild-type and IL-17-deficient mice transplanted with AX cells in the bone marrow cavity.

A hole was created in the femur at the distal side with a 26G needle in mice under general anesthesia and a total of $5x10^5$ AX cells was injected into the bone marrow cavity of indicated mice. Shown is analysis of survival in each group (each n=6). IL-17-deficient mice transplanted in the bone marrow cavity with AX cells exhibited significantly prolonged survival relative to similarly transplanted wild-type mice.

Supplemental Figure S3. Detection of IL-17A expression in CD4-positive cells in wild-type but not IL-17-deficient mice.

Eight-week-old wild-type or IL-17-deficient mice were transplanted subcutaneously with AX cells, and a month later, frozen tumor sections were prepared and stained with rat anti-CD4 or rabbit anti IL-17A followed by Alexa488 or Alexa594-conjugated anti-rat Ig' or Alexa594-conjugated anti-rabbit Ig', respectively. Nuclei were DAPI-stained and cells were observed under a fluorescence microscopy (a). The percentage of IL-17A-positive cells/CD4-positive cells in sections from both genotypes was determined in randomly selected 200-μm squares (b). Graph indicates mean percentage of IL-17A-positive cells/CD4-positive cells (%) ± s.d. (each n=15, *p<0.05). Bar,100 μm.

Supplemental Figure S4. IL-17 signaling inhibits osteoblastic differentiation of human osteosarcoma MG63 cells.

Human osteosarcoma MG63 cells were stimulated 24 hours with various concentrations of human IL-17A (10~1000 ng/ml) and the number of AX cells analyzed using an MTT assay based on absorbance at 570 nm. Data represent mean absorbance ± s.d. (n=6, *p<0.05). (b) MG63 cells were cultured 24 h in the presence (IL-17A) or absence (vehicle) of IL-17A (500ng/ml), total RNA was prepared, and expression of *ALP*, *Osteocalcin* (*Oc*) or *Runx2* relative to *β-actin* was analyzed by quantitative real-time PCR. Data represent mean expression of *ALP, Oc* or *Runx2* relative to *β-actin* ± s.d. (n=6, *p<0.05).

Supplemental Figure S5. IL-17 signaling inhibits mineralization of AX cells.

Parental-, SCR-, IL-17RA KO1- or IL17RA KO2- AX cells were cultured 14 days in osteogenic medium with (IL-17A) or without (vehicle) IL-17A (500ng/ml), and mineralized nodules were stained with Alizarin Red S (a). Alizarin Red S was then extracted in 10% acetic acid and assessed for absorbance at 405 nm to quantify mineralization. Data represent mean mineralization in the IL-17A group, as determined by absorbance at 405 nm, relative to that in the vehicle group ± s.d. (n=6, *p<0.05).

Supplemental Figure S6. IL-17RA expression is deleted on the cell surface of IL-17RA splenocytes.

Splenocytes were isolated from wild-type or IL-17RA-deficient mice, stained with anti-IL-17RA or ISO type control antibody, and analyzed by flow cytometry (left). Data represent mean IL-17RA expression on IL-17RA-deficient splenocytes relative to that on wild-type splenocytes ± s.d. (right) (n=6, *p<0.05).

Supplemental Figure S7. IL17RA is expressed in osteosarcoma biopsy samples from patients.

Total RNA was extracted from 33 biopsy samples from human osteosarcoma patients, and microarray analysis performed to detect *IL-17RA*, *IL-17A* and *IL-17F*. Data represent mean *IL-17RA*, *IL-17A* or *IL-17F* expression relative to *Actin* ± s.d. (n=33, *p<0.05).

Figure S8. Full-length western blots.

Full-length western blots of data shown in Fig. 4c. First antibodies were anti-ALP (a), anti-Osteocalcin (b), anti-Runx2 (c) and anti-Actin (d). Arrows show bands of indicated proteins. Similarly, Fig. 5d is also shown in (e) - (h).

Supplemental Figures

Supplemental Figure S1.

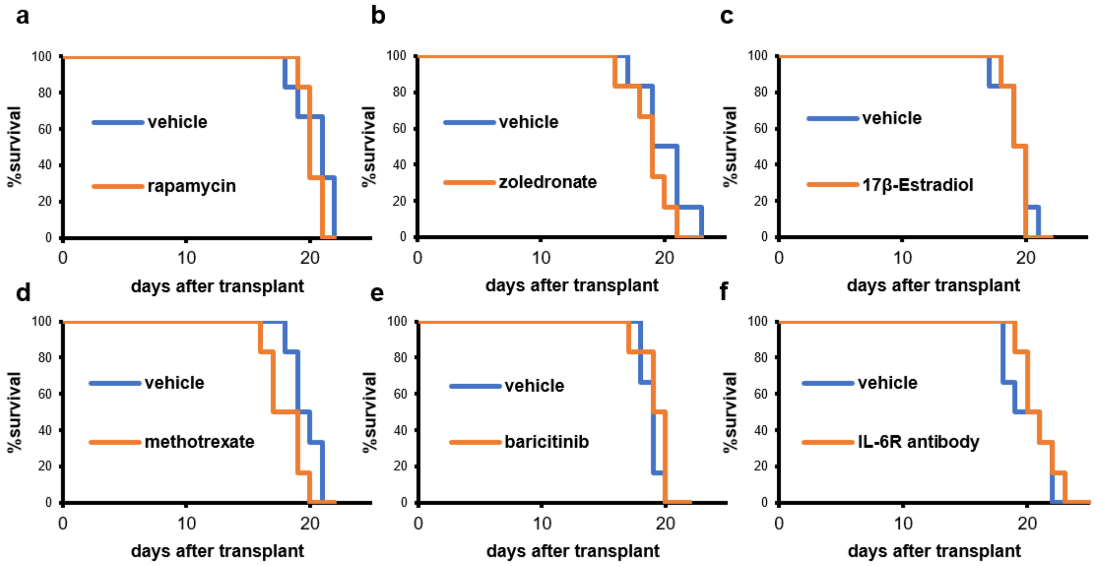

Supplemental Figure S2.

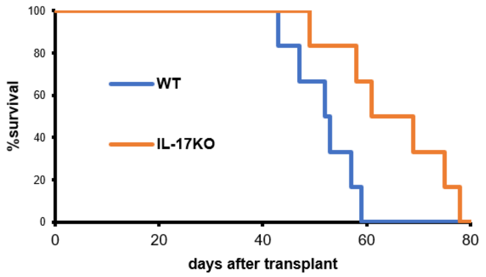

Supplemental Figure S3.

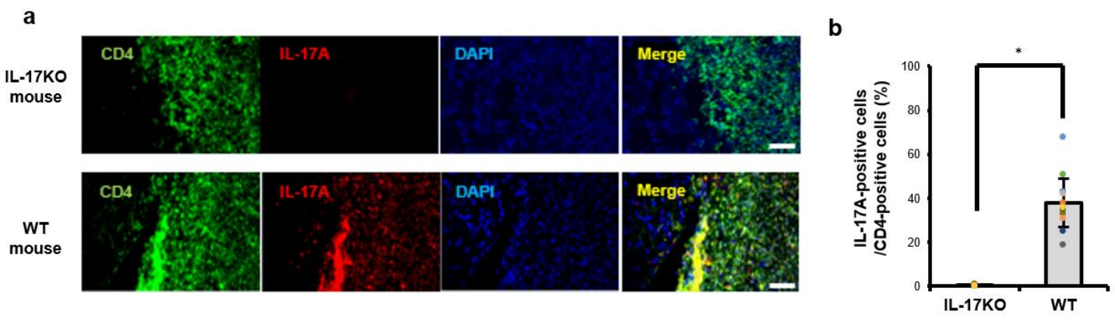

Supplemental Figure S4.

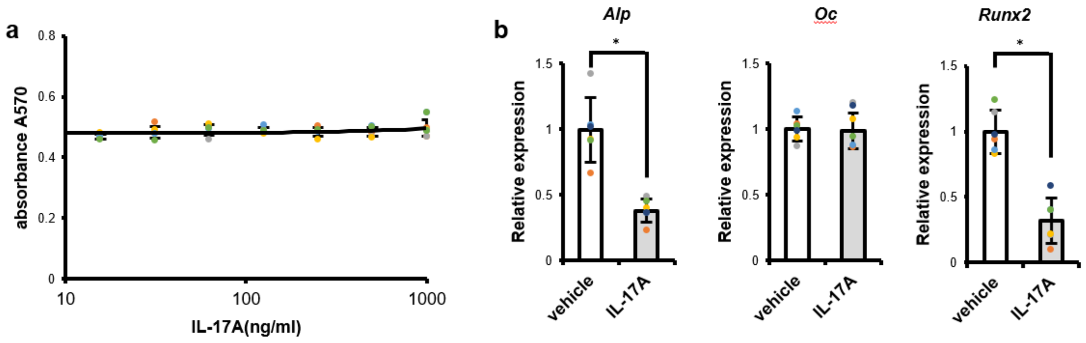

Supplemental Figure S5.

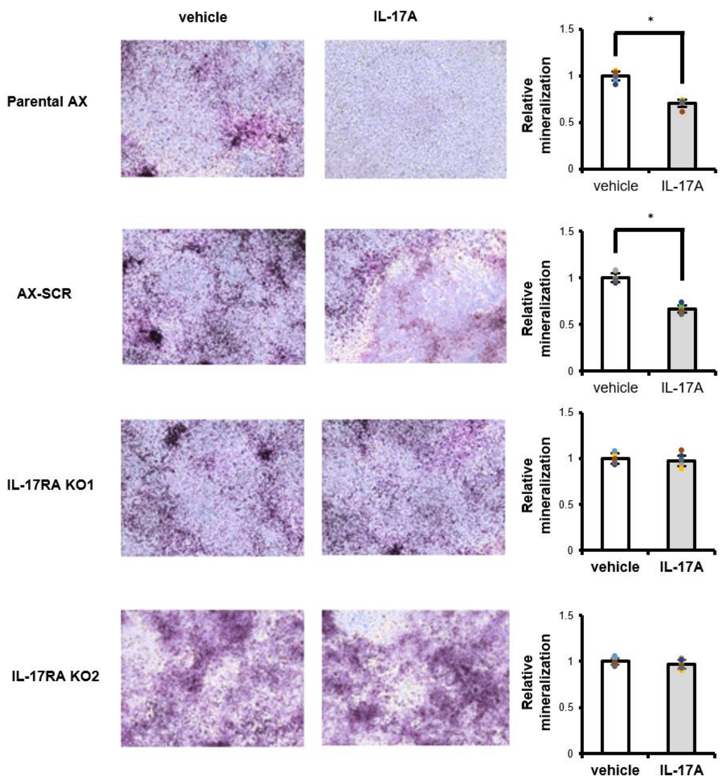

Supplemental Figure S6.

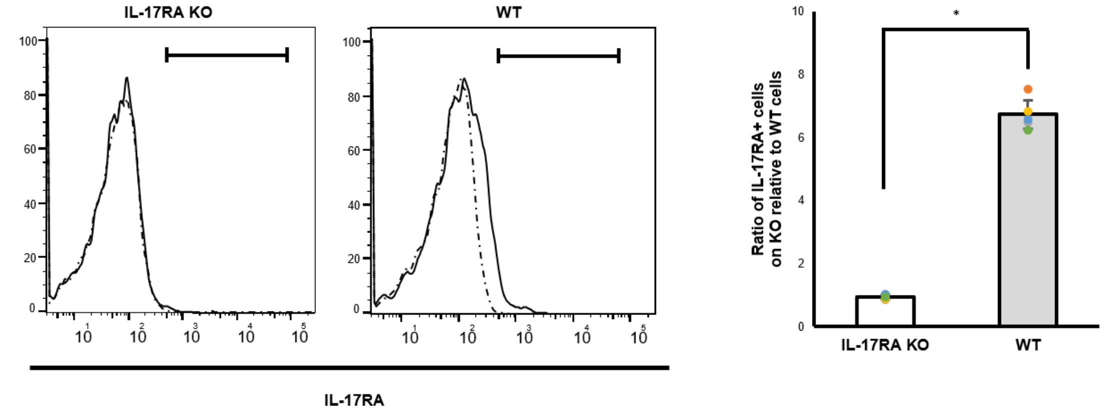

Supplemental Figure S7.

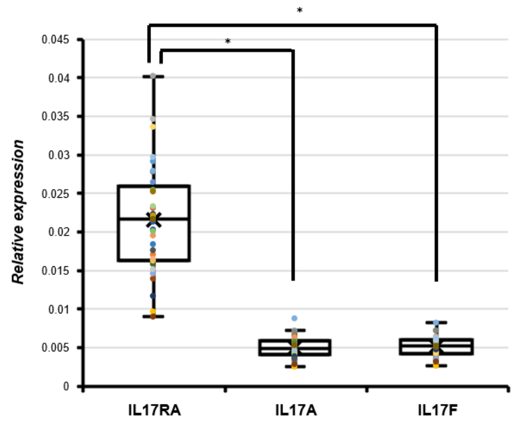

Figure S8.

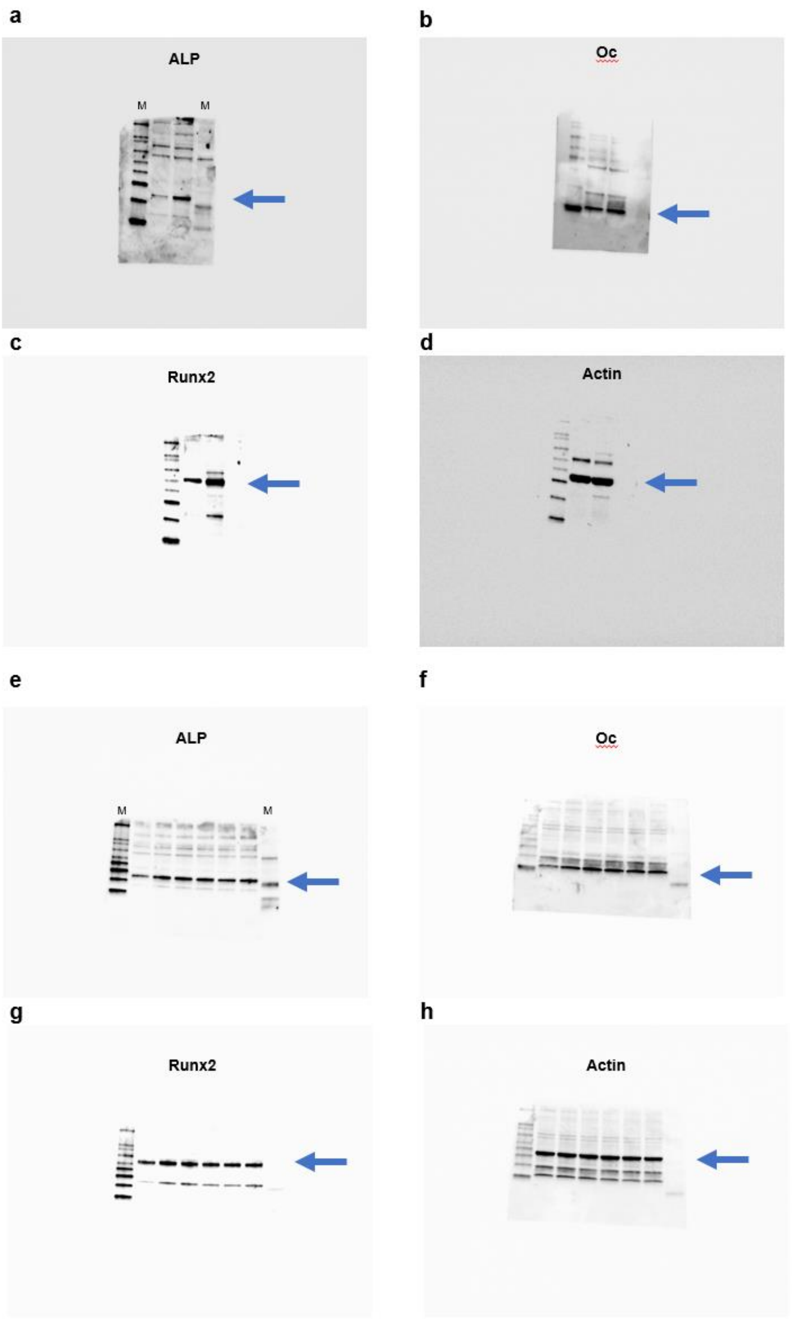

Supplement: Supplementary file 1 — Supplementary Figures. [file 41598_2023_49016_MOESM1_ESM.pdf]
